# Minimally invasive liver surgery for hepatocellular carcinoma in patients with portal hypertension

**DOI:** 10.1093/bjsopen/zrad037

**Published:** 2023-04-28

**Authors:** Daniel Aliseda, Gabriel Zozaya, Pablo Martí-Cruchaga, Juan Lujan, Ana Almeida, Nuria Blanco, Lucas Sabatella, Bruno Sangro, Fernando Rotellar

**Affiliations:** Department of General Surgery, Clinica Universidad de Navarra, University of Navarra, Pamplona, Spain; Department of General Surgery, Clinica Universidad de Navarra, University of Navarra, Pamplona, Spain; Institute of Health Research of Navarra (IdisNA), Pamplona, Spain; Department of General Surgery, Clinica Universidad de Navarra, University of Navarra, Pamplona, Spain; Institute of Health Research of Navarra (IdisNA), Pamplona, Spain; Department of General Surgery, Clinica Universidad de Navarra, University of Navarra, Pamplona, Spain; Department of General Surgery, Clinica Universidad de Navarra, University of Navarra, Pamplona, Spain; Department of General Surgery, Clinica Universidad de Navarra, University of Navarra, Pamplona, Spain; Department of General Surgery, Clinica Universidad de Navarra, University of Navarra, Pamplona, Spain; Institute of Health Research of Navarra (IdisNA), Pamplona, Spain; Liver Unit and Hepato-Pancreato-Biliary Oncology Area, Clinica Universidad de Navarra and CIBEREHD, Pamplona, Spain; Department of General Surgery, Clinica Universidad de Navarra, University of Navarra, Pamplona, Spain; Institute of Health Research of Navarra (IdisNA), Pamplona, Spain

## Introduction

For patients with early stage hepatocellular carcinoma (HCC), liver resection is a mainstay of curative treatment. Patients with a solitary tumour, Child–Pugh A cirrhosis and serum bilirubin of 1 mg/dl are considered ideal candidates for liver resection^1,2^. For patients with portal hypertension, current guidelines recommend careful consideration of liver resection based on the hierarchical interaction of portal hypertension, liver function and resection extent^1,3^. Open liver resection has been used in the majority of published studies on liver resection and portal hypertension. Although there is limited published experience of minimally invasive liver resection (MILR), using MILR in these patients appears to be associated with favourable outcomes^4^. Particularly in patients with Child–Pugh A cirrhosis, but also in patients with more advanced cirrhosis^5^, MILR offers significant advantages in the surgical treatment of HCC including reduced intraoperative bleeding, fewer complications and minimized surgical aggression, which improves recovery^6,7^. If these benefits are also found in patients with portal hypertension, MILR may represent a step forward in the surgical treatment of patients with HCC and portal hypertension.

This systematic review and meta-analysis aimed to summarize the intraoperative, postoperative and survival outcomes of MILR in patients with HCC and portal hypertension.

## Methods

This systematic review was conducted according to the PRISMA guidelines and registered in the PROSPERO (CRD42022300797) platform^8^. Using a rigorous search strategy (see *Supplementary material* and *Table S1*), three electronic databases (PubMed, MEDLINE (via Ovid) and Scopus) were searched from database inception to 28 December 2022. Inclusion criteria were studies including adults aged ≥ 18 years with a diagnosis of HCC undergoing laparoscopic, robotic or hybrid liver surgery and with underlying portal hypertension. Clinically significant portal hypertension (CSPH) was defined as a transjugular hepatic venous pressure gradient (HVPG) ≥10 mmHg, and indirect signs of portal hypertension were defined as thrombocytopenia (<100 000 platelets/mm^3^) and splenomegaly or the presence of oesophageal varices at endoscopy. Studies were excluded if platelet count alone or HVPG <10 mmHg was used for the assessment of portal hypertension. Reviews, editorials and case reports with fewer than five patients were also excluded. Data extraction and methodological assessment of the studies are presented in the *Supplementary material*. Two independent meta-analyses, a pooled meta-analysis of means and proportions and a patient-level survival data meta-analysis were performed to assess perioperative and survival outcomes respectively. A detailed description of the statistical analysis and outcomes of interest can be found in the *Supplementary material* (pages 5–6).

## Results

Six studies met the inclusion criteria (*Table S2*). *Figure S1* and *Table S3* show the PRISMA 2020 flowchart and the methodological quality assessment, respectively.

### Perioperative outcomes pooled meta-analysis

For the meta-analysis of perioperative outcomes, five reports^9–11,13,14^ including 168 patients met the inclusion criteria. Baseline patient and tumour characteristics are shown in *Table S4*. According to the Child–Pugh classification, 147 patients were in class A (87.5 per cent) and 21 were in class B (12.5 per cent). Portal hypertension was defined as HVPG ≥10 mmHg in two studies^11,13^.

One hundred and sixty-five purely laparoscopic surgeries were performed along with three hybrid surgeries^13^. Minor resections (one or two Couinaud’s segments) were performed in 138 patients (82.1 per cent). Sixty-five wedge resections accounted for 38.6 per cent of all surgeries, followed by 70 segmentectomies or bisegmentectomies (41.7 per cent) and three left lateral sectionectomies (1.8 per cent). Three studies^9–11^ included major resections (three to four Couinaud’s segments), including 14 hemihepatectomies and 16 anatomical resections of three segments. The mean operative time was 197.6 min (95 per cent c.i. 186.3–208.9) (*I*^2^ = 93.0 per cent; *P* < 0.005) (*Fig. S2a*). The amount of intraoperative blood loss was 285.1 ml (95 per cent c.i. 246.7–323.4) (*I*^2^ = 75.1 per cent; *P* = 0.003) (*Fig. S2b*). The transfusion rate was reported in three studies^10,11,14^ with a proportion of 11 per cent (95 per cent c.i. 2.0–24.0 per cent) (*I*^2^ = 51.9 per cent; *P* = 0.13) (*Fig. S2c*). The conversion rate was reported in four studies^9–11,14^ with a rate of 6 per cent (95 per cent c.i. 2.0–11.0 per cent) (*I*^2^ = 0.0 per cent; *P* = 0.48) (*Fig. S2d*). The operative details are summarized in *Table S5*.

### Postoperative outcomes

A summary of the postoperative outcomes is shown in *Table S6*. The mean duration of hospital stay was 8 days (95 per cent c.i. 7.6–8.5) (*I*^2^ = 97.8 per cent; *P* < 0.005) (*Fig. S3a*). The overall morbidity rate was 38 per cent (95 per cent c.i. 28.0–50.0) (*I*^2^ = 45.6 per cent; *P* = 0.12) (*Fig. S3b*). The major complication rate (Clavien–Dindo ≥ III) was 10 per cent (95 per cent c.i. 5.0–15.0) (*I*^2^ = 0.0 per cent; *P* = 0.95) (*Fig. S3c*). The comprehensive complication index was 9.3 (95 per cent c.i. 7.2–11.5) (*I*^2^ = 0.3 per cent; *P* = 0.404) (*Fig. S3d*). None of the studies reported mortality during the first 90 days after surgery. R0 resection was reported in four studies ^9–11,13^ and was achieved at 95 per cent (95 per cent c.i. 90.0–98.0) (*I*^2^ = 0.0 per cent; *P* = 0.59) (*Fig. S4*).

### Liver-specific complications

The overall rate of postoperative haemorrhage was 2 per cent (95 per cent c.i. 0.0–7.0 per cent) (*I*^2^ = 10.3 per cent; *P* = 0.34) (*Fig. S5a*). All patients who experienced postoperative haemorrhage required transfusion, but no surgical or invasive intervention was necessary in any patient. The proportion of liver failure was 5 per cent (95 per cent c.i. 1.0–11.0 per cent) (*I*^2^ = 0.0 per cent; *P* = 0.59) (*Fig. S5b*) with one patient requiring admission to the ICU and the rest of the patients not requiring invasive interventions. The postoperative ascites rate was 7 per cent (95 per cent c.i. 1.0–16.0 per cent) (*I*^2^ = 39.9 per cent; *P* = 0.17) (*Fig. S5c*) with more than 90 per cent of patients exhibiting transient ascites treated with diuretics. Two studies^11,13^ reported on unresolved liver decompensation (3 months after surgery), with a rate of 5 per cent (95 per cent c.i. 0.0–14.0 per cent) (*I*^2^ = 0.0 per cent) (*Fig. S5d*). These patients were managed with medical treatment without the need for salvage transplantation. Finally, three studies reported that 50 per cent of all Clavien–Dindo ≥ 3 complications were due to liver-specific complications, which are summarized in *Table S7*.

### Patient-level survival data meta-analysis

**Fig. 1:**
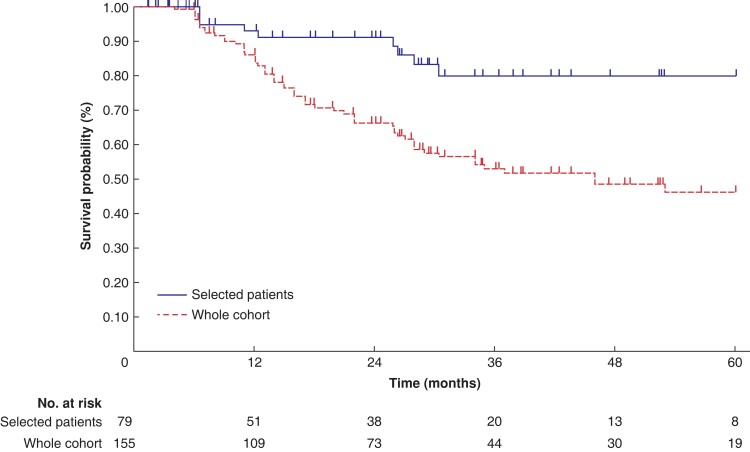
Kaplan–Meier overall survival curves and number-at-risk table for the whole cohort and for selected patients with hepatocellular carcinoma and portal hypertension who underwent minimally invasive liver resection

Five studies^9,10,12–14^ were included in the survival analysis, including 155 patients. Unadjusted overall survival (OS) rates at 1, 3 and 5 years were 86.1 per cent (95 per cent c.i. 78.8–91.0), 53.0 per cent (95 per cent c.i. 43.3–61.9) and 46.3 per cent (95 per cent c.i. 35.7–56.2) respectively (*Fig. 1*). However, in selected patients (the majority of Child–Pugh A cirrhosis, tumours < 5 cm and excluding advanced HCC stages), the unadjusted OS was 93.0 per cent (95 per cent c.i. 82.4–97.3), 79.9 per cent (95 per cent c.i. 64.0–89.3) and 79.9 per cent (95 per cent c.i. 64.0–89.3) at 1, 3 and 5 years respectively (*Fig. 1*). Four studies reported^10,12–14^ HCC recurrence after a mean follow-up of 24.4 (±8.0) months. The tumour recurrence rate was 31 per cent (95 per cent c.i. 15.0–49.0) (*I*^2^ = 61.1 per cent; *P* = 0.05) (*Fig. S6*). The detailed survival outcomes are shown in *Table S8*.

## Discussion

In patients with HCC and portal hypertension, MILR demonstrates reaching optimal rates of intraoperative and postoperative outcomes with minimal liver-specific complications. In addition, the results are similar to current benchmarks for laparoscopic liver surgery^15^. Furthermore, this study suggests that in well-selected patients, MILR achieves excellent long-term OS outcomes.

MILR is associated with less liver mobilization, better preservation of perihepatic collateral circulation, the pneumoperitoneum assisting effect and limitation of insensible fluid loss secondary to small incisions, among others^16,17^. These properties are particularly relevant in cirrhotic patients in whom MILR has been shown to decrease blood loss and complications, such as ascites and posthepatectomy liver failure^6,7^. The advantages of MILR over open liver resection are particularly relevant for patients with portal hypertension. Most of the modest published results in these patients are based on open liver resection. Performing laparoscopy in HCC patients with portal hypertension has already shown better postoperative outcomes compared with open surgery, as well as being an independent factor to achieve textbook outcomes^4,14,18^. In addition to the intra- and postoperative benefits, MILR may also offer advantages in the surgical management of recurrence. In the case of repeat liver resection or salvage liver transplantation, MILR reduces severe adhesions, making repeat hepatectomy or retransplantation safer^19^.

Therefore, the results observed in this meta-analysis suggest that MILR in HCC patients with portal hypertension is a safe, effective and promising approach. If supported by more robust studies and in well-selected patients, MILR could represent a significant advance towards the curative treatment of patients with HCC and portal hypertension.

## Supplementary Material

zrad037_Supplementary_Data

## Data Availability

Data to reproduce this report is available in the published studies.
