# Supplementary material for: Minimally invasive liver surgery for hepatocellular carcinoma in patients with portal hypertension
Source: BJS Open. 2023 Apr 28;7(2):zrad037. doi: 10.1093/bjsopen/zrad037 (PMC10146929; doi:10.1093/bjsopen/zrad037)
Supplement: zrad037_Supplementary_Data [file zrad037_supplementary_data.docx]

**MINIMALLY INVASIVE LIVER SURGERY FOR HEPATOCELLULAR CARCINOMA IN PATIENTS WITH PORTAL HYPERTENSION: A GAME CHANGER ARRIVES?**

Daniel Aliseda MD^1^, Gabriel Zozaya MD^1,2^, Pablo Martí-Cruchaga MD^1,2^, Juan Lujan MD^1^, Ana Almeida MD^1^, Nuria Blanco MD^1^, Lucas Sabatella MD^1^, Bruno Sangro MD, PhD^2,3^, Fernando Rotellar MD, PhD^1,2^.

1.- Department of General Surgery, Clinica Universidad de Navarra, University of Navarra, Av. Pío XII, 36, 31008, Pamplona, Spain.

2.- Institute of Health Research of Navarra (IdisNA), Pamplona, Spain.

3.- Liver Unit and HPB Oncology Area, Clinica Universidad de Navarra and CIBEREHD, Pamplona, Spain.

**Corresponding author**

Daniel Aliseda. Department of General Surgery, Clinica Universidad de Navarra, University of Navarra, Av. Pío XII, 36, 31008, Pamplona, Spain. Phone number: 34 948255400-ext 4711-. e-mail: dalisedaj@unav.es

**Supplementary Materials - Index**

[SEARCH STRATEGY, DATA EXTRACTION AND STUDY METHODOLOGICAL QUALITY ASSESSMENT 2](#_Toc128650064)

[Search strategy 2](#_Toc128650065)

[Data extraction 2](#_Toc128650066)

[Study methodological quality assessment 2](#_Toc128650067)

[STATISTICAL ANALYSIS 5](#_Toc128650068)

[Pooled proportions meta-analysis 5](#_Toc128650069)

[Reconstruction of individual patient survival data and survival analysis 5](#_Toc128650070)

[STUDIED VARIABLES 6](#_Toc128650071)

[STUDY LIMITATIONS 6](#_Toc128650072)

[ANALYSIS OF PUBLICATION BIAS 7](#_Toc128650073)

[PATIENT-LEVEL SURVIVAL DATA RECONSTRUCTION 21](#_Toc128650074)

[Guo et al (2022)^1^ 21](#_Toc128650075)

[Zheng et al (2020)^2^ 22](#_Toc128650076)

[Lim et al (2019)^4^ 23](#_Toc128650077)

[Molina et al (2018)^5^ 24](#_Toc128650078)

[Harada et al (2016)^6^ 25](#_Toc128650079)

[SUPPLEMENTARY FIGURES 26](#_Toc128650080)

[SUPPLEMENTARY TABLES 32](#_Toc128650081)

[REFERENCES 42](#_Toc128650082)

# SEARCH STRATEGY, DATA EXTRACTION AND STUDY METHODOLOGICAL QUALITY ASSESSMENT

## Search strategy

The electronic databases (PubMed, MEDLINE (via Ovid) and Scopus) were reviewed combining Mesh terms and Boolean operators. The following search strategy was conducted to identify relevant studies: "hepatocellular carcinoma OR hepatocarcinoma OR HCC" AND "portal hypertension OR clinically significant portal hypertension " AND "surgery OR resection OR hepatectomy". An additional search was performed by cross-referencing. The final search was conducted on 28 December 2022 and was performed from database inception.

The literature search retrieved a total of 1.947 studies. After discarding duplicates, record screening was performed for 1.250 articles from the electronic searches. A full text review of 31 studies was conducted. Rejected articles as well as the reason for non-inclusion are summarized in Table 1S (Supplementary material). Finally, 6 studies met the inclusion criteria^1–6^ (Table 2S). The PRISMA 2020 flowchart is presented in Figure 1S.

## Data extraction

Two investigators (D.A and N.B) performed the first blinded screening in duplicate. Reading the full text, also in duplicate (D.A and N.B), helped to determine which articles should be finally selected. Rejected articles were accurately identified during the selection and the reasons for non-compliance for inclusion were properly noted. In case of disagreements, the decision was settled by group consensus. Using a data extraction form created especially for the review, data was extracted blindly, in duplicate (D.A. and L.S.) and cross-checked. The following components were at least extracted from each research: study period, baseline patient and tumor characteristics, intraoperative parameters, postoperative complications (including specific liver complications), as well as long-term overall and disease-free survival.

## Study methodological quality assessment

The quality of the selected studies was assessed by D.A and N.B using the Methodological Index for Non-Randomized Studies (MINORS) criteria^7^. This tool assesses methodological quality by taking into account 8 domains in the case of non-comparative studies and adds 4 more domains in case of comparative studies.

All 6 included studies were observational and comparative (Table 2S): five retrospective and one prospective study were included. The MINORS score assessment for each study is shown in the Supplementary Appendix (Table 3S).


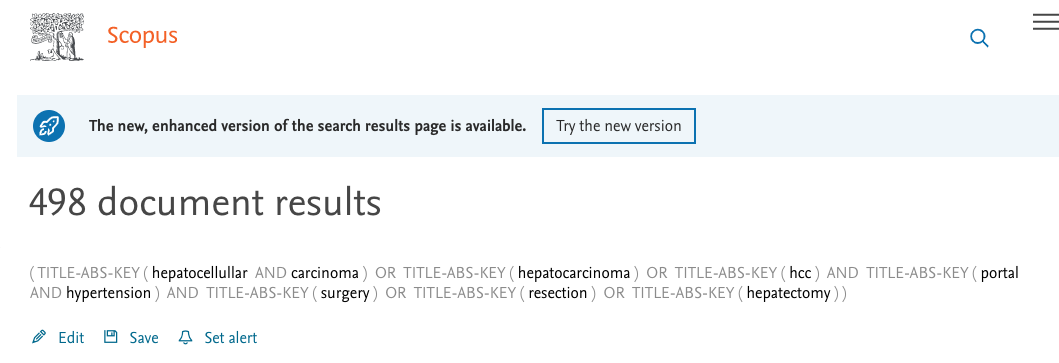


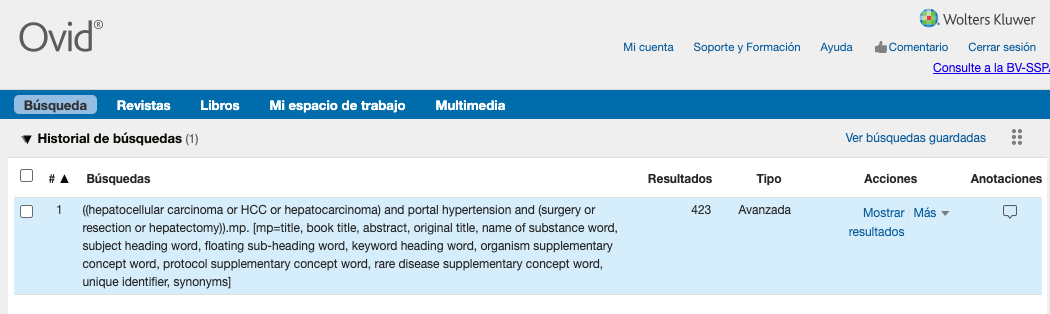


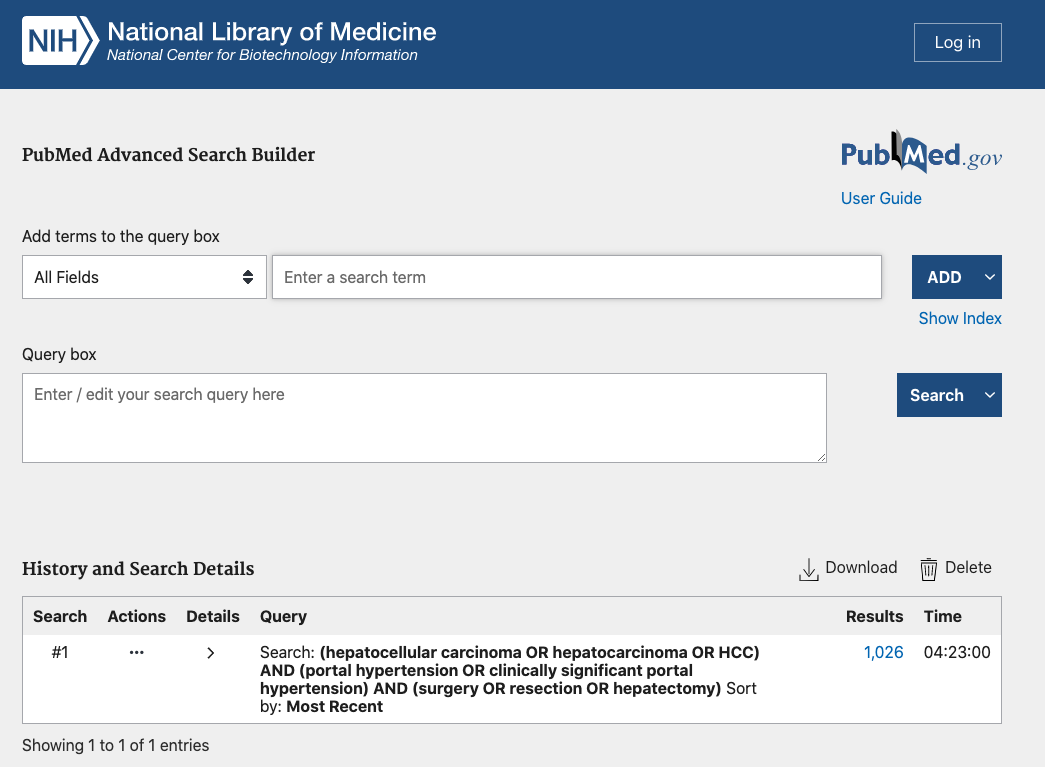


# STATISTICAL ANALYSIS

Two meta-analyses, using pooled and patient-level survival data, were performed to analyze perioperative and survival outcomes respectively.

## Pooled proportions meta-analysis

Firstly, a pooled meta-analysis was performed using the STATA program “metan” and “metaprop” to pool means and proportions, respectively. For the studies only reporting the median and range (or interquartile range), the formulas proposed by *Luo et* al and *Wan et al* ^8,9^ were used to calculate mean values and standard deviation, respectively. To perform a meta-analysis of binomial data and simultaneously avoid a biased pooled estimate derived from studies excluded from the analysis (near to 0 or 1), we utilized the exact binomial method to calculate the individual study confidence intervals and the Freeman-Tukey double arcsine for the transformation of the proportions with variance stabilization^10^. The derived pooled proportions were calculated using random-effect models. For the pooled means meta-analysis, a common-effect inverse-variance model was applied. Cochran's Q test was used to measure the heterogeneity between studies, and considerable significance was determined according to DerSimonian and Laird's technique when the p value was less than 0.10. The fraction of variability across studies was measured using the Higgins statistic (I^2^). Low, moderate, and high heterogeneity were characterized as I^2^ values of 25, 50, and 75%, respectively^11^. Publication bias was investigated using funnel plots. All tests were two-sided with a significance level of 0.05. This meta-analysis was conducted with STATA version 16 (StataCorp, College Station, Texas 77845 USA).

## Reconstruction of individual patient survival data and survival analysis

To summarize more precisely the survival information, we extracted the OS data from the published curves (with the Digitizelt). Once the step function values and periods of the steps were extracted, the individual patient survival data was inferred using the iterative algorithm based on the Kaplan Meier estimation method proposed by Guyot et al ^12–14^. All the survival data extracted were checked against the original data (number at risk and 1 to 5- year OS rates). The Kaplan-Meier method was used to create the graphs with the extracted survival data and to calculate the unadjusted OS.

# STUDIED VARIABLES

*1. Short-term outcomes:*

- Intraoperative parameters: conversion, blood loss, transfusion rate and operative time.
- Post-operative outcomes: length of hospital stay, post-operative morbidity (according to Clavien-Dindo^15^and CCI^16^), R0 resection and 90-day mortality.
- Liver specific complications: Liver failure (according to the International Study Group of Liver Surgery (ISGLS) or the “50–50 criteria”^17,18^), hemorrhage, ascites and unresolved liver decompensation (defined as jaundice, ascites, or encephalopathy 3 months after surgery)

*2. Long-term outcomes:* Overall survival (months since MILR).

# STUDY LIMITATIONS

The significant heterogeneity in the assessment of certain outcomes such as operative time, intraoperative blood loss or hospital stay is a limitation of the study. Given the variability in surgical techniques and postoperative protocols between centers, this heterogeneity is to be expected. Publication bias also exists for some variables, probably due to the recent introduction of the MILR in patients with PHT. Furthermore, only 2 of the included studies ^3,5^ defined CSPH invasively by measuring HVPG, as recommended by current guidelines, which is a limitation as indirect signs of PHT lacks sensitivity and specificity compared to HVPG measurement ^19–21^. The majority of MILRs have been performed by experienced surgeons in centers with extensive experience. Most series are retrospective. The impact of selection bias is unknown, and most studies have limited numbers of patients included.

# ANALYSIS OF PUBLICATION BIAS

All studies were independently assessed for bias in each variable using funnel plots. Publication bias was found for operative time and length of hospital stay (Supplementary material S1 and S5).

**OPERATIVE TIME**

(Supplementary material. 1)

Funnel plot showing publication bias; operative time (minutes) (X-axis) with it is standard error (Y-axis)

**CONVERSION RATE**

(Supplementary material. 2)

Funnel plot showing publication bias; conversion rate (%) (X-axis) with it is standard error (Y-axis)

**BLOOD LOSS**

(Supplementary material. 3)

Funnel plot showing publication bias; blood loss (ml) (X-axis) with it is standard

error (Y-axis)

**TRANSFUSION**

(Supplementary material. 4)

Funnel plot showing publication bias; transfusion rate (%) (X-axis) with it is standard error (Y-axis)

**LENGTH OF HOSPITAL STAY**

(Supplementary material. 5)

Funnel plot showing publication bias; hospital stay (days) (X-axis) with it is standard error (Y-axis)

**OVERALL MORBIDITY**

(Supplementary material.6)

Funnel plot showing publication bias; overall morbidity (%) (X-axis) with it is standard error (Y-axis)

**MAJOR COMPLICATIONS (CLAVIEN-DINDO≥ 3)**

(Supplementary material. 7)

Funnel plot showing publication bias; major complications (%) (X-axis) with it is standard error (Y-axis)

**CCI**

(Supplementary material. 8)

Funnel plot showing publication bias; CCI (X-axis) with it is standard error (Y-axis)

**R0 RESECTION**

(Supplementary material. 9)

Funnel plot showing publication bias; R0 resection (%) (X-axis) with it is standard error (Y-axis)

**RECURRENCE**

(Supplementary material. 10)

Funnel plot showing publication bias; recurrence rate (%) (X-axis) with it is standard error (Y-axis)

**POSTOPERATIVE HEMORRAGHE**

(Supplementary material.11)

Funnel plot showing publication bias; postoperative hemorrhage rate (%) (X-axis) with it is standard error (Y-axis)

**POSTOPERATIVE LIVER FAILURE**

(Supplementary material.12)

Funnel plot showing publication bias; postoperative liver failure rate (%) (X-axis) with it is standard error (Y-axis)

**POSTOPERATIVE ASCITES**

(Supplementary material.13)

Funnel plot showing publication bias; 46e liver failure rate (%) (X-axis) with it is standard error (Y-axis)

**LIVER DECOMPENSATION**

(Supplementary material.14)

Funnel plot showing publication bias; unresolved liver decompensation rate (%) (X-axis) with it is standard error (Y-axis)

# PATIENT-LEVEL SURVIVAL DATA RECONSTRUCTION

## Guo et al (2022)^1^

Kaplan Meier curve from original paper


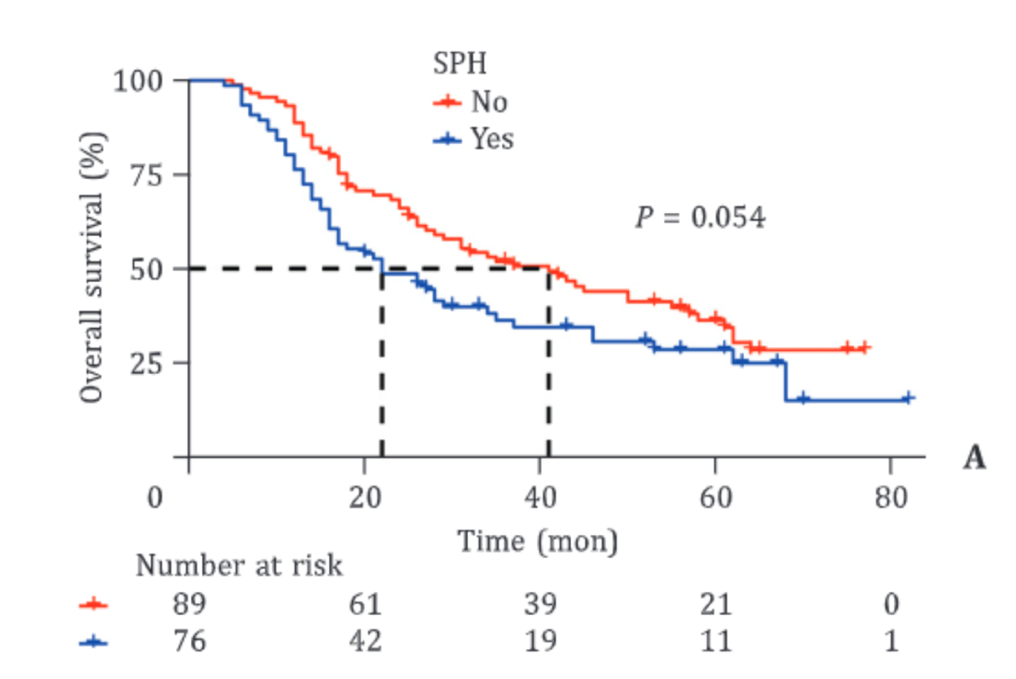


Reconstructed survival curves - including number-at-risk tables-.

## Zheng et al (2020)^2^

Kaplan Meier curve from original paper


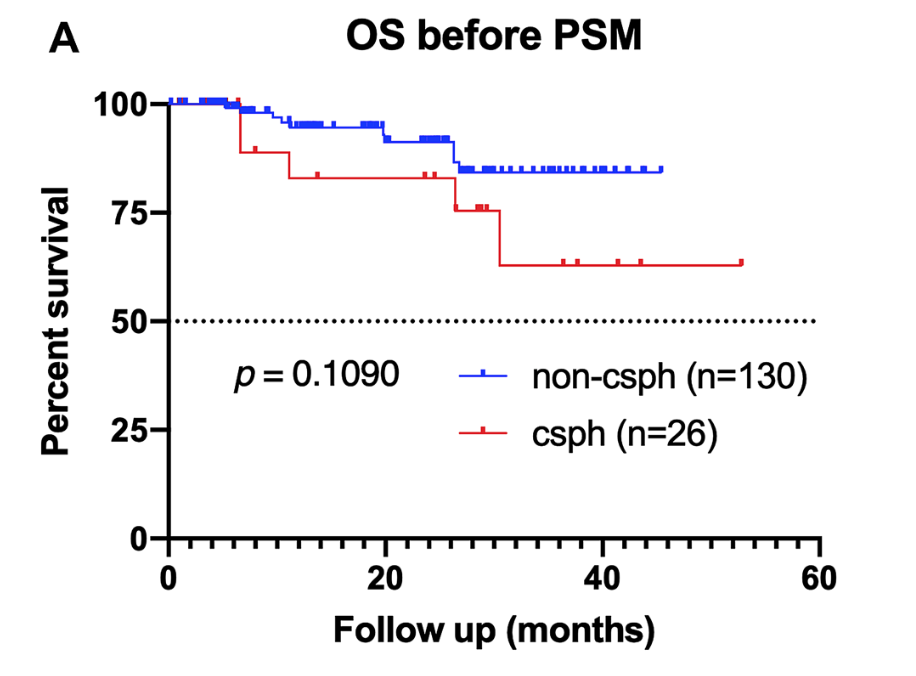


Reconstructed survival curves - including number-at-risk tables-.

## Lim et al (2019)^4^

Reconstructed survival curves - including number-at-risk tables-.

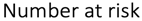

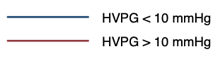


## Molina et al (2018)^5^

Kaplan Meier curve from original paper


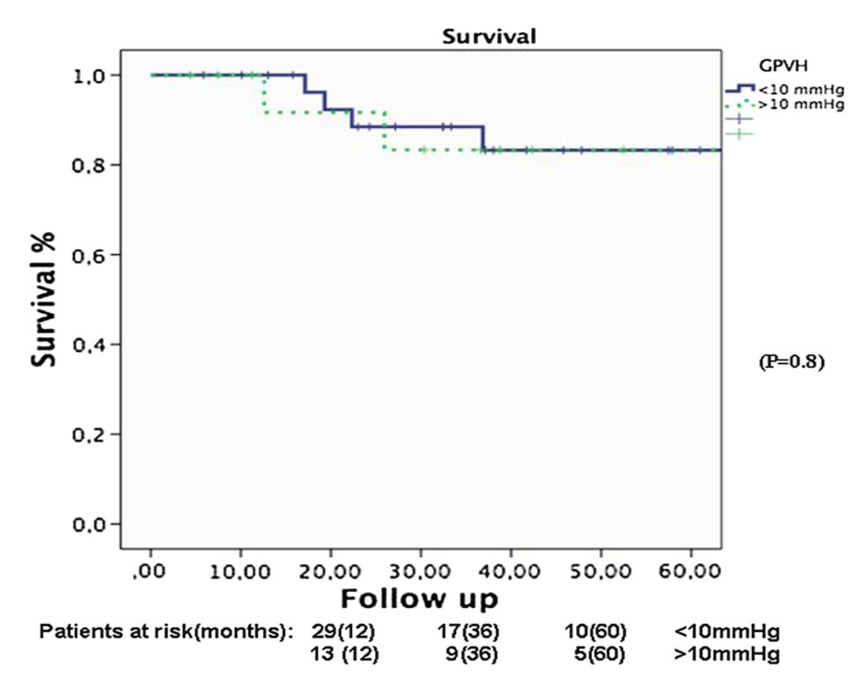


Reconstructed survival curves - including number-at-risk tables-.

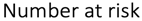


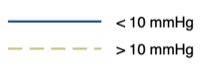


­­

## Harada et al (2016)^6^

Kaplan Meier curve from original paper


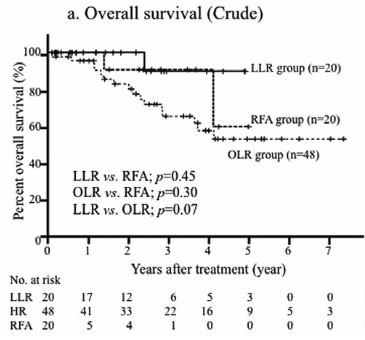


Reconstructed survival curves - including number-at-risk tables-.

# SUPPLEMENTARY FIGURES


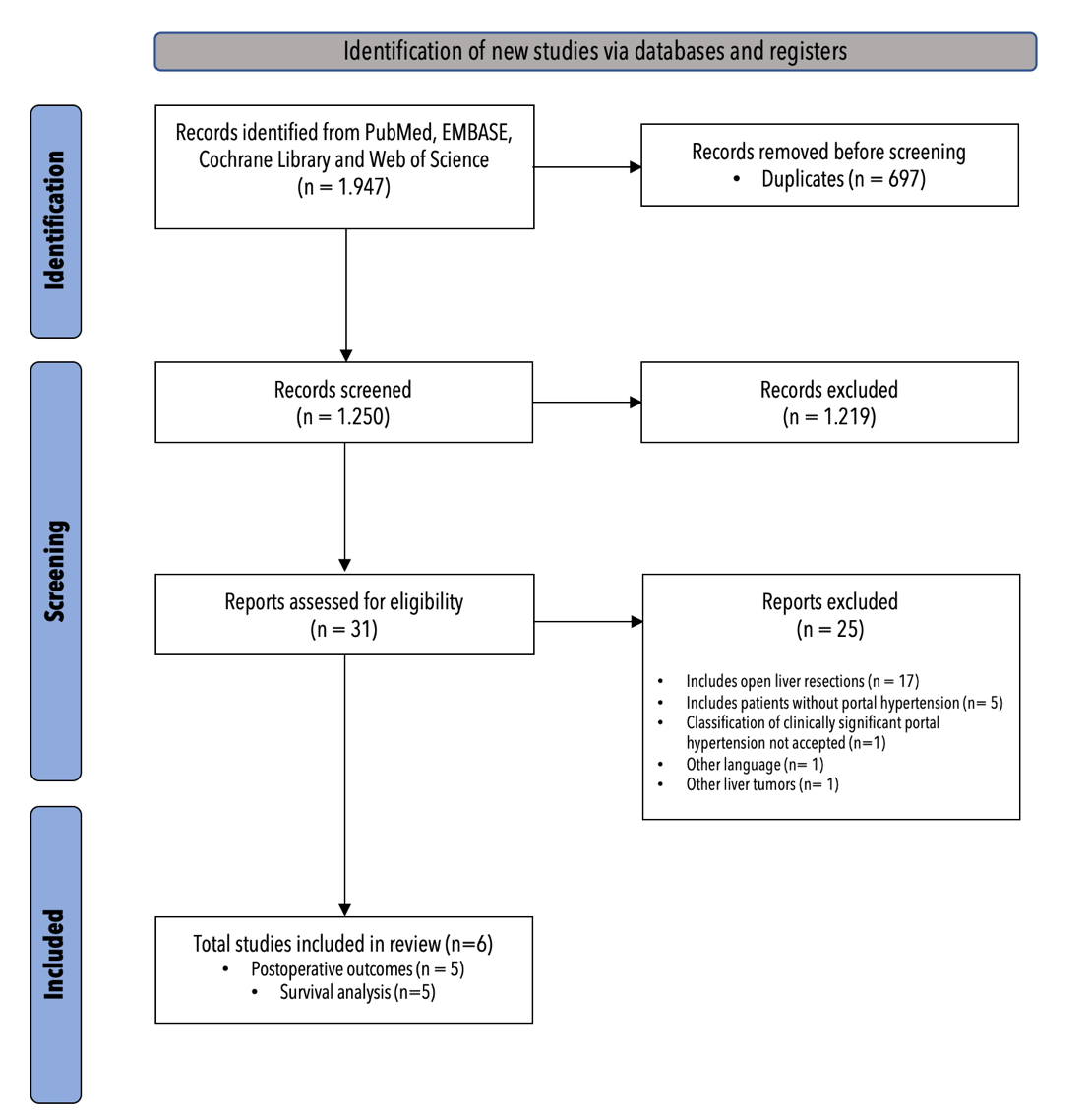


**Figure S1.** PRISMA flowchart

**
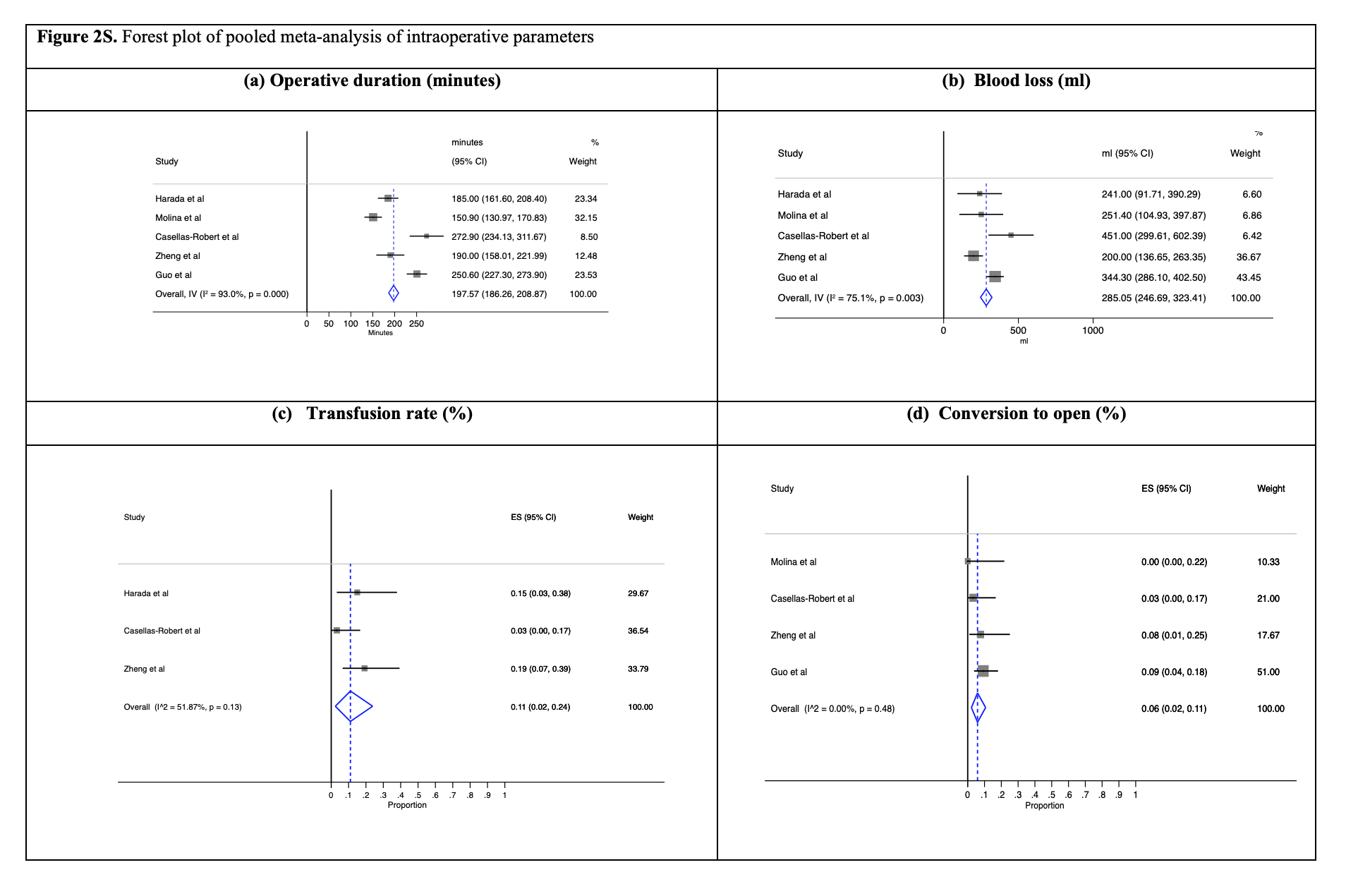
**

**
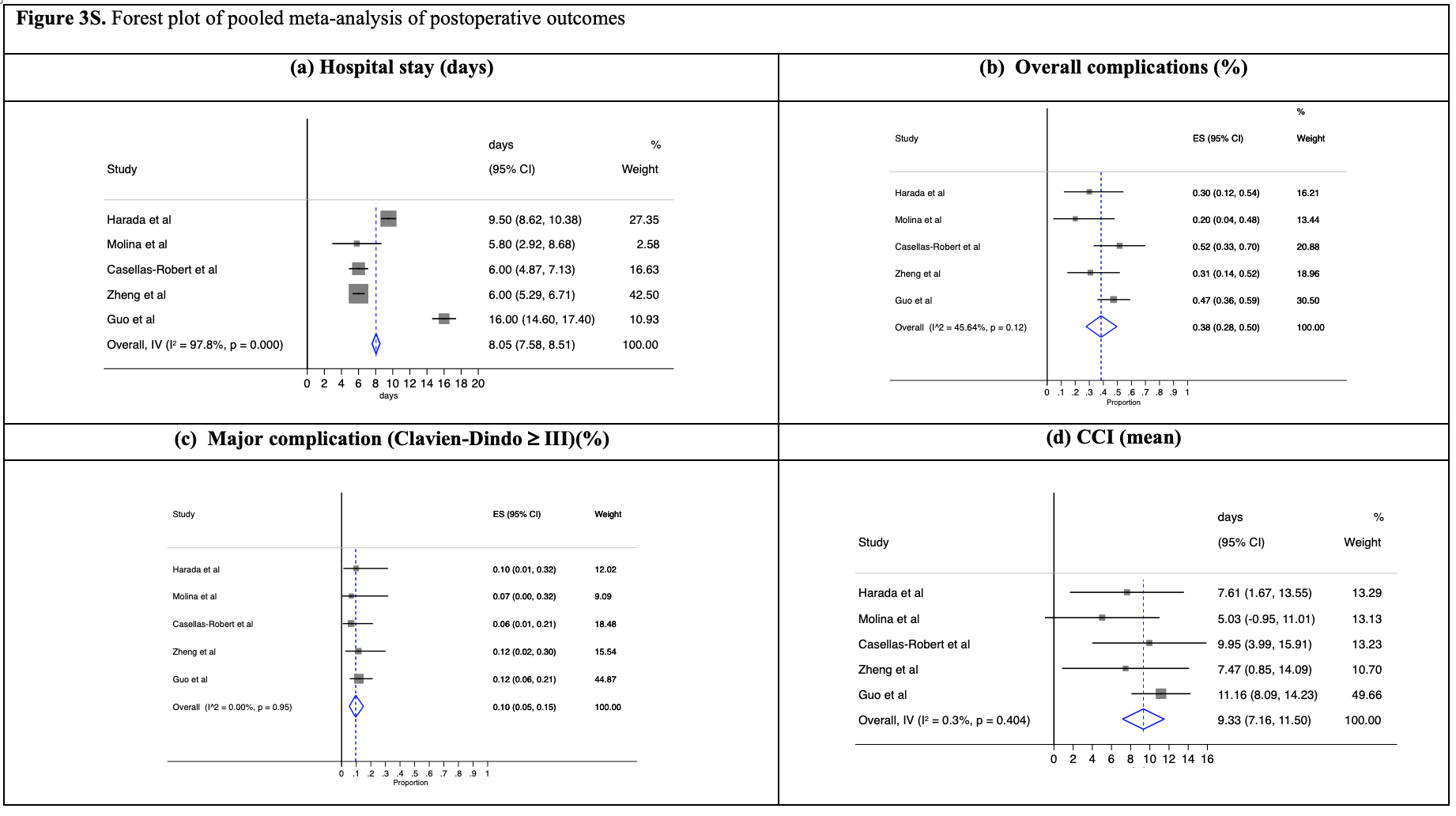
**

**Figure S4.** Forest plot of pooled meta-analysis for R0 resection

**
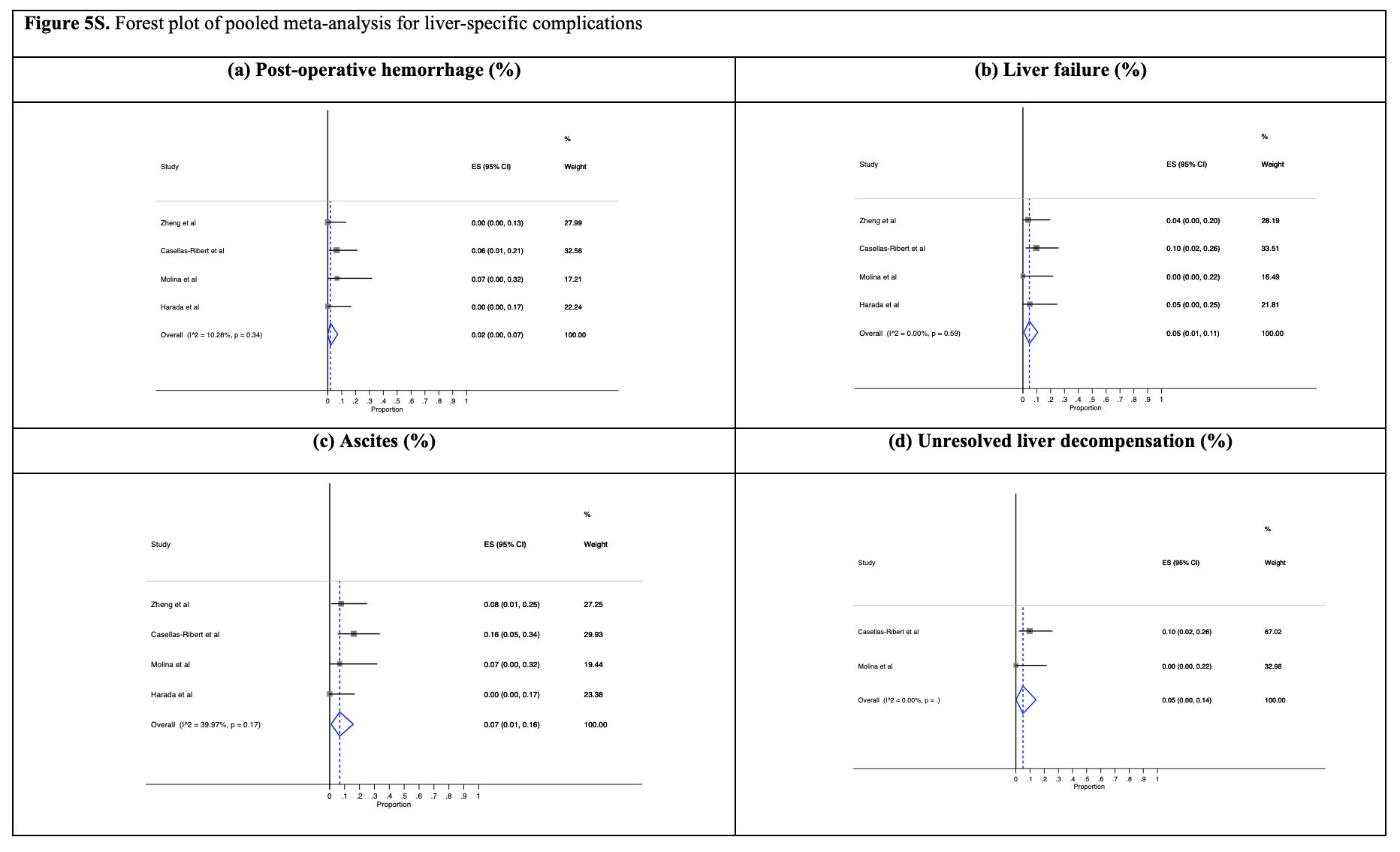
**

**Figure S6.** Forest plot of pooled meta-analysis for recurrence rate

# SUPPLEMENTARY TABLES

| **Table S1.** Studies excluded from the meta-analysis after full text review | | |
| --- | --- | --- |
| **PMID** | **Study** | **Cause of exlusion** |
| **34838392** | Impact of concurrent splenectomy and esophagogastric devascularization on surgical outcomes of partial hepatectomy for hepatocellular carcinoma in patients with clinically significant portal hypertension: A multicenter propensity score matching analysis | Includes open liver resection |
| **33711132** | Laparoscopic and open liver resection for hepatocellular carcinoma with Child-Pugh B cirrhosis: multicentre propensity score-matched study | Includes patients without portal hypertension |
| **33294830** | Liver resection for hepatocellular carcinoma in patients with clinically significant portal hypertension | Includes open liver resection |
| **32707003** | Laparoscopic Liver Surgery: What Are the Advantages in Patients with Cirrhosis and Portal Hypertension? Systematic Review and Meta-Analysis with Personal Experience | Includes patients without portal hypertension |
| **32274662** | Minimally Invasive Versus Open Liver Resection for Hepatocellular Carcinoma in the Setting of Portal Vein Hypertension: Results of an International Multi-institutional Analysis | Classification of clinically significant portal hypertension not accepted |
| **31702672** | The preoperative platelet to albumin ratio predicts the prognosis of hepatocellular carcinoma patients without portal hypertension after liver resection | Includes open liver resection |
| **30990885** | Hepatectomy for hepatocellular carcinoma after perioperative management of portal hypertension | Includes open liver resection |
| **30849786** | Albumin-Indocyanine Green Evaluation (ALICE) grade combined with portal hypertension to predict post-hepatectomy liver failure | Includes open liver resection |
| **30758604** | [Partial liver resection vs. transarterial chemoembolization for hepatocellular carcinoma beyond the Milan criteria and portal hypertension] | Other language |
| **30572742** | Portal hypertension predicts short-term and long-term outcomes after hepatectomy in hepatocellular carcinoma patients | Includes open liver resection |
| **28717870** | Laparoscopic liver resections for hepatocellular carcinoma. Can we extend the surgical indication in cirrhotic patients? | Includes patients without portal hypertension |
| **28261694** | Impact of clinically significant portal hypertension on surgical outcomes for hepatocellular carcinoma in patients with compensated liver cirrhosis: a propensity score matching analysis | Includes open liver resection |
| **28058552** | Complications after Hepatectomy for Hepatocellular Carcinoma Independently Shorten Survival: A Western, Single-Center Audit | Includes patients without portal hypertension |
| **26911609** | Comparison of the Outcomes of Patients with Hepatocellular Carcinoma and Portal Hypertension After Liver Resection Versus Radiofrequency Ablation | Includes open liver resection |
| **26911609** | Laparoscopic hepatectomy in cirrhotics: safe if you adjust technique | Includes other liver tumors |
| **26059406** | Surgical Resection for Hepatocellular Carcinoma with Concomitant Esophageal Varices | Includes open liver resection |
| **25958066** | Resection versus ablation in hepatitis B virus-related hepatocellular carcinoma patients with portal hypertension: A propensity score matching study | Includes open liver resection |
| **25886495** | The role of clinically significant portal hypertension in hepatic resection for hepatocellular carcinoma patients: a propensity score matching analysis | Includes open liver resection |
| **25700311** | Hepatic Resection for Hepatocellular Carcinoma in Patients With Portal Hypertension | Includes open liver resection |
| **25457251** | A preliminary study on surgery for hepatocellular carcinoma patients with portal hypertension | Includes open liver resection |
| **25268959** | Hepatic Resection Is Safe and Effective for Patients with Hepatocellular Carcinoma and Portal Hypertension | Includes open liver resection |
| **25225514** | Comparison of liver resection and transplantation for Child-pugh A cirrhotic patient with very early hepatocellular carcinoma and portal hypertension | Includes open liver resection |
| **31828498** | Laparoscopic versus open limited liver resection for hepatocellular carcinoma with liver cirrhosis: a propensity score matching study with the Hiroshima Surgical study group of Clinical Oncology (HiSCO) | Includes patients without portal hypertension |
| **26325538** | Hepatic venous pressure gradient in the preoperative assessment of patients with resectable hepatocellular carcinoma | Includes open liver resection |
| **25700311** | Hepatic Resection for Hepatocellular Carcinoma in Patients With Portal Hypertension | Includes open liver resection |

| Table S2. Selected studies reporting minimally invasive liver surgery for patients with hepatocellular carcinoma and portal hypertension | | | | | | |
| --- | --- | --- | --- | --- | --- | --- |
| Author | **Year of publication** | **Study period** | **Country** | **No. patients** | **Study design** | **Centre** |
| Guo et al^1^ | 2022 | Jan 2013 - April 2018 | China | 76 | Retrospective | Multi |
| Zheng et al.^2^ | 2020 | Feb 2016 - Sept 2019 | China | 26 | Retrospective | Single |
| Lim et al^4^ | 2019 | Jan 2014 - Jan 2018 | France | 18 | Prospective | Single |
| Casellas-Robert et al.^3^ | 2020 | April 2011 - March 2018 | Spain | 31 | Retrospective | Multi |
| Molina et al.^5^ | 2017 | Dec 2005 - Apri 2016 | Spain | 15 | Retrospective | Single |
| Harada et al.^6^ | 2016 | Jan 2008 - Sept 2015 | Japan | 20 | Retrospective | Multi |

| Table S3. Methodological items for non-randomized studies (MINORS) score for studies included | | | | | | | | | | | | | |
| --- | --- | --- | --- | --- | --- | --- | --- | --- | --- | --- | --- | --- | --- |
| Author | **A clearly stated aim** | **Inclusion of consecutive patients** | **Prospective collection of data** | **Endpoints appropriate to the aim of the study** | **Unbiased assessment of the study endpoint** | **Follow-up period appropriate to the aim of the study** | **Loss to follow up less than 5%** | **Prospective calculation of the study size** | **An adequate control group** | **Contemporary groups** | **Baseline equivalence of groups** | **Adequate statistical analyses** | **Total score** |
| Guo et al^1^ | 2 | 2 | 0 | 2 | 0 | 2 | 2 | 0 | 2 | 2 | 2 | 2 | **18** |
| Zheng et al.^2^ | 2 | 2 | 0 | 2 | 0 | 2 | 1 | 0 | 2 | 2 | 2 | 2 | **17** |
| Lim et al^4^ | 2 | 2 | 2 | 2 | 0 | 2 | 2 | 1 | 2 | 2 | 2 | 2 | **21** |
| Casellas-Robert et al.^3^ | 2 | 2 | 0 | 2 | 0 | 2 | 2 | 0 | 2 | 2 | 2 | 2 | **18** |
| Molina et al.^5^ | 2 | 2 | 0 | 2 | 0 | 2 | 2 | 0 | 2 | 2 | 2 | 2 | **18** |
| Harada et al.^6^ | 2 | 2 | 0 | 2 | 0 | 2 | 2 | 0 | 2 | 2 | 2 | 2 | **18** |

| Table S4. Preoperative characteristics of HCC patients with portal hypertension undergoing MILR | | | | | | | | | | | | | | | |
| --- | --- | --- | --- | --- | --- | --- | --- | --- | --- | --- | --- | --- | --- | --- | --- |
| Author | **Age (years)** | **Sex ratio (M/F)** (%) | **BMI (kg/m^2^)** | **Child Pugh A/B/C** | **ASA score ^a^** | **AST** **(IU/L)** | **ALT** **(IU/L)** | **Albumin** **(g/dL)** | **Bilirubin** **(mg/dL)** | **Protombin time** (**% of normal)** | **HVPG (mmHg)** | **Platelet count (10^5^/mm^3^)** | **AFP (ng/ml)** | **Solitary HCC** ^b^ | **Tumor size (cm)** |
| Guo et al^1^ | 50.4 ± 5.3 | 79/21 | 23.2 ± 3.0 | 62/14/0 | 2 (2-3) | NR | 41.5 (25.0-64.0) ^c^ | 40.32 ± 4.07 | 13.60 (8.15-20.30) ^c^ | NR | NR | 92.0 (69.0-126.8) ^c^ | 129.35 (14.78-1129.50) ^c^ | 54 (71.1) | ≤ 3 cm: 43 (56.6%) |
| Zheng et al.^2^ | 58.9 ± 9 | 88/12 | 23.9 ± 3.2 | 19/7/0 | 2 (1-3) | 37.2 ± 28.1 | 37.8 ± 23.1 | 3.5 ± 0.4 | NR | 14.5 ± 1.3 ^+^ | NR | 74.8 ± 52.7 | 274.4 ± 543.5 | 21 (80.8) | 2.61 ± 1 |
| Lim et al^4^ | 65.1 (8.5) | 61/39 | 28.2 (7.7) | 18/0/0 | 2 (1-3) | 47.4 (18.9) | 39.8 (22.2) | 3.7 ± 0.5 | 0.77 (0.42 | 86% (10.2) | 15.26 ± 4.4 | 113.2 ± 33.2 | 151.5 ± 254.2 | 16 (88.8) | 2.6 ± 0.25* |
| Casellas-Robert et al.^3^ | 64 ± 8 | 64/36 | 28.3 ± 4.7 | 31/0/0 | 2 (1-4) | 42.8 ± 20.1 | 34.7 ± 17 | 4 ± 0.5 | 0.67 ± 0.3 | NR | 12.7 ± 3.1 | NR | 9 ± 7.7 | 28 (90.3) | 1.9 ± 1.5* |
| Molina et al.^5^ | 63.5 ± 7.2 | 73/27 | 26.2 ± 3.2 | 15/0/0 | NR | 96.5 ± 63 | 72.4 ± 36.5 | NR | 0.95 ± 0.5 | 84% ± 12 | 11.34 ± 1.15 | 154.6 ± 58.4 | NR | NR | 2.7 ± 1.3* |
| Harada et al.^6^ | 74 ± 6 | 45/50 | NR | 20/0/0 | NR | 43 ± 16 | 41 ± 17 | 4.2 ± 0.5 | 0.9 ± 0.3 | 91 ± 11 | NR | 120 ± 60 | 41 ± 53 | 19 | 1.8 ± 0.6 |
| *Data are expressed as mean (standard deviation or range) unless otherwise specified.*  *Abbreviations:* *HCC*, hepatocellular carcinoma; *BMI,* body mass index; *ASA,* American Society of anaesthesiologist; *AST*, Aspartate transaminase; *ALT* Alanine transaminase; *HVPG* hepatic venous pressure gradient; *AFP,* Alpha-fetoprotein; *MILR,* minimally invasive liver resection*; NR,* data not reported  *^a^ Median (range);* ^b^ no. (%); *Median (interquartile range)* ^c^  **Histopathology; + seconds* | | | | | | | | | | | | | | | |

| Table S5. Operative characteristics of HCC patients with portal hypertension undergoing MILR | | | | | | | | | |
| --- | --- | --- | --- | --- | --- | --- | --- | --- | --- |
| Author | **Minor resections** | **Major resections** | **Operative time (min)^a^** | **Pringle manoeuvre** | **Inflow clamping (min)^a^** | **Surgical technique** | **Blood loss (ml)** ^a^ | **Blood transfusion** | **Conversion** |
| Guo et al^1^ | 49 (64.5) | 27 (35.5) | 250.6 (102) | 41 (53.9) | NR | 20WR +8 LH + 3RH + 45 AR | 344.3 (254.7) | NR | 7 (9.2) |
| Zheng et al.^2^ | 24 (92.3) | 2 (7.7) | 190.4 (79.2) | 13 (50.0) | 29.7 (15.7) | 9 WR + 3SEC + 12SEG +2hH | 200 (156.86) | 5 (19.2) | 2 (7.7) |
| Casellas-Robert et al.^3^ | 30 (96.8) | 1 (3.2) | 272.9 (105.7) | 25 (80.6) | 37.1 (31.1) | 16WR + 14 SEG +1RH | 451 (412.7) | 1 (3.2) | 1 (3.2) |
| Molina et al.^5^ | 15 (100) | 0 (0.0) | 150.9 (36) | 6 (40) | 18.9 (4.3) | 3WR + 8SEG + 4BS | 251.4 (264.5) | NR | 0 (0.0) |
| Harada et al.^6^ | 20 (100) | 0 (0.0) | 185 (50) | NR | NR | 17 WR + 3 SEG | 241 (319) | 3 | NR |
| *Data are expressed as n (%) unless otherwise specified.*  *Abbreviations: HCC*, hepatocellular carcinoma; *WR,* wedge resection; *SEC,* left lateral sectionectomy*; SEG,* segmentectomy*; BS,* bisegmentectomy*; RH,* right hepatectomy*; LH,* left segmentectomy*; hH,* hemihepatectomy*; AR: Anatomical resections; LOS,* length of stay; *NR,* data not reported;  *MILR,* minimally invasive liver resection  ^a^ *Mean (standard deviation)* | | | | | | | | | |

| Table S6. Postoperative outcomes of HCC patients with portal hypertension undergoing MILR | | | | | | | | |
| --- | --- | --- | --- | --- | --- | --- | --- | --- |
| Author | **LOS (day)** *^b^* | **Morbidity** | **Clavien-Dindo (I-II)** | **Clavien-Dindo (≥3)** | **CCI** | **Reoperation** | **Mortality (90 days)** | **R0 resection** |
| Guo et al^1^ | 16 (6.05) | 36 (47.4) | 27 (35.5) | 9 (11.8) | 11.16 (13.44) | NR | 0 (0.0) | 71 (93.4) |
| Zheng et al.^2^ | 6 (1.76) | 8 (30.1) | 5 (19.2) | 3 (11.5) | 7.47 (16.4) | 0 (0.0) | 0 (0.0) | 25 (96.2) |
| Casellas-Robert et al.^3^ | 6 (3.1) | 16 (51.6) | 14 (45.2) | 2 (6.5) | 9.95 (16.24) | NR | 0 (0.0) | 28 (90.3) |
| Molina et al.^5^ | 5.8 (5.2) | 3 (20.0) | 2 (13.3) | 1 (6.7) | 5.03 (10.8) | 1 (6.7) | 0 (0.0) | 15 (100) |
| Harada et al.^6^ | 9.5 (1.9) | 6 (30.0) | 4 (20.0) | 2 (10) | 7.61 (12.7) | 0 (0.0) | 0 (0.0) | NR |
| *Data are expressed as n (%) unless otherwise specified.*  *Abbreviations: HCC*, hepatocellular carcinoma; *LOS,* length of stay; *NR,* data not reported; *MILR,* minimally invasive liver resection  ^a^ *Mean (standard deviation) ^b^ Median (range)* | | | | | | | | |

| Table S7. Postoperative liver specific complications of HCC patients with portal hypertension undergoing MILR | | | | |
| --- | --- | --- | --- | --- |
| Author (year) | **Haemorrhage** | **Liver failure** | **Ascites** | **Unresolved liver decompensation** |
| Guo et al^1^ | NR | NR | NR | NR |
| Zheng et al.^2^ | 0 (0.0) | 1 (3.8) | 2 (7.6) | NR |
| Casellas-Robert et al.^3^ | 2 (7.0) | 3 (10.0) | 5 (16.0) | 3 (10.0) |
| Molina et al.^5^ | 1 (6.7) | 0 (0.0) | 1 (6.7) | 0 (0.0) |
| Harada et al.^6^ | 0 (0.0) | 1 (5.0) | 0 (0.0) | NR |
| *Data are expressed as n (%) unless otherwise specified.*  *MILR,* minimally invasive liver resection*;* NR*, not reported* | | | | |

| Table S8. Survival outcomes of HCC patients with portal hypertension undergoing MILR | | | | | | | | | | | |
| --- | --- | --- | --- | --- | --- | --- | --- | --- | --- | --- | --- |
| Author (year) | **No. patients** | **Follow up (months)** *^b^* | **Recurrence** | *1-year OS* | *2-year OS* | *3-year OS* | *5-year OS* | *1-year DFS* | *2-year DFS* | *3-year DFS* | *5-year DFS* |
| Guo et al^1^ | 76 | 61 | NR | 80.3% | NR | 36.2% | 30.6% | 69.5% | NR | 33.5% | 18.6% |
| Zheng et al.^2^ | 26 | 19.6 (0.2–40.6) | 7 (26.9) | 83.0%, | 75.4% | 62.9% | NR | 76.4% | 64.2% | 57.7% | NR |
| Lim et al^4^ | 18 | 18 (1–42) | 2 (11) | 100% | 100% | NR | NR | 94% | 79% | NR | NR |
| Molina et al.^5^ | 15 | 38 (7–100) | 8 (53.3) | 100% | 92.2% | 83.9% | 83.9% | 87.8% | NR | 61.4% | NR |
| Harada et al.^6^ | 20 | NR | 8 (40.0) | 100% | 100% | 90.3% | 90.3% | 91.8% | 66.8% | 44.7% | 29.6% |
| *Data are expressed as n (%) unless otherwise specified.*  *Abbreviations: HCC*, hepatocellular carcinoma; *MILR,* minimally invasive liver resection; *OS*, overall survival; *DFS,* disease free survival.  ^a^ *Mean (standard deviation) ^b^ Median (range)* | | | | | | | | | | | |

# REFERENCES

1 Guo Z-Y, Hong Y, Tu B, Cheng Y, Wang X-M. Laparoscopic liver resection for hepatocellular carcinoma complicated with significant portal hypertension: A propensity score-matched survival analysis. *Hepatob Pancreat Dis*. 2022;

2 Zheng J, Feng X, Liang Y, Cai J, Shi Z, Kirih MA, *et al.* Safety and feasibility of laparoscopic liver resection for hepatocellular carcinoma with clinically significant portal hypertension: a propensity score-matched study. *Surg Endosc*. 2021; 35: 3267–3278.

3 Casellas-Robert M, Lim C, Lopez-Ben S, Lladó L, Salloum C, Codina-Font J, *et al.* Laparoscopic Liver Resection for Hepatocellular Carcinoma in Child–Pugh A Patients With and Without Portal Hypertension: A Multicentre Study. *World J Surg*. 2020; 44: 3915–3922.

4 Lim C, Osseis M, Lahat E, Doussot A, Sotirov D, Hemery F, *et al.* Safety of laparoscopic hepatectomy in patients with hepatocellular carcinoma and portal hypertension: interim analysis of an open prospective study. *Surg Endosc*. 2019; 33: 811–820.

5 Molina V, Sampson-Dávila J, Ferrer J, Fondevila C, Gobbo RD del, Calatayud D, *et al.* Benefits of laparoscopic liver resection in patients with hepatocellular carcinoma and portal hypertension: a case-matched study. *Surg Endosc*. 2018; 32: 2345–2354.

6 Harada N, Maeda T, Yoshizumi T, Ikeda T, Kayashima H, Ikegami T, *et al.* Laparoscopic Liver Resection Is a Feasible Treatment for Patients with Hepatocellular Carcinoma and Portal Hypertension. *Anticancer Res*. 2016; 36: 3489–3497.

7 Slim K, Nini E, Forestier D, Kwiatkowski F, Panis Y, Chipponi J. Methodological index for non‐randomized studies (MINORS): development and validation of a new instrument. *Anz J Surg*. 2003; 73: 712–716.

8 Luo D, Wan X, Liu J, Tong T. Optimally estimating the sample mean from the sample size, median, mid-range, and/or mid-quartile range. *Stat Methods Med Res*. 2018; 27: 1785–1805.

9 Wan X, Wang W, Liu J, Tong T. Estimating the sample mean and standard deviation from the sample size, median, range and/or interquartile range. *Bmc Med Res Methodol*. 2014; 14: 135.

10 Nyaga VN, Arbyn M, Aerts M. Metaprop: a Stata command to perform meta-analysis of binomial data. *Archives Public Heal*. 2014; 72: 39.

11 Higgins JPT, Thompson SG. Quantifying heterogeneity in a meta‐analysis. *Stat Med*. 2002; 21: 1539–1558.

12 Guyot P, Ades A, Ouwens MJ, Welton NJ. Enhanced secondary analysis of survival data: reconstructing the data from published Kaplan-Meier survival curves. *Bmc Med Res Methodol*. 2012; 12: 9.

13 Tierney JF, Stewart LA, Ghersi D, Burdett S, Sydes MR. Practical methods for incorporating summary time-to-event data into meta-analysis. *Trials*. 2007; 8: 16.

14 Wei Y, Royston P. Reconstructing Time-to-event Data from Published Kaplan–Meier Curves. *Stata J*. 2017; 17: 786–802.

15 Dindo D, Demartines N, Clavien P-A. Classification of Surgical Complications. *Ann Surg*. 2004; 240: 205–213.

16 Slankamenac K, Graf R, Barkun J, Puhan MA, Clavien P-A. The Comprehensive Complication Index. *Ann Surg*. 2013; 258: 1–7.

17 Balzan S, Belghiti J, Farges O, Ogata S, Sauvanet A, Delefosse D, *et al.* The “50-50 Criteria” on Postoperative Day 5. *Ann Surg*. 2005; 242: 824–829.

18 Rahbari NN, Garden OJ, Padbury R, Brooke-Smith M, Crawford M, Adam R, *et al.* Posthepatectomy liver failure: A definition and grading by the International Study Group of Liver Surgery (ISGLS). *Surgery*. 2011; 149: 713–724.

19 Franchis R de, Bosch J, Garcia-Tsao G, Reiberger T, Ripoll C, Faculty BV, *et al.* BAVENO VII - RENEWING CONSENSUS IN PORTAL HYPERTENSION Report of the Baveno VII Consensus Workshop: personalized care in portal hypertension. *J Hepatol*. 2021;

20 Cucchetti A, Cescon M, Golfieri R, Piscaglia F, Renzulli M, Neri F, *et al.* Hepatic venous pressure gradient in the preoperative assessment of patients with resectable hepatocellular carcinoma. *J Hepatol*. 2016; 64: 79–86.

21 Qamar AA, Grace ND, Groszmann RJ, Garcia-Tsao G, Bosch J, Burroughs AK, *et al.* Platelet count is not a predictor of the presence or development of gastroesophageal varices in cirrhosis. *Hepatology*. 2008; 47: 153–159.
